# Carotid Stump Syndrome: A Case That Highlights the Necessity of Digital Subtraction Angiography for the Prompt Management of the Syndrome

**DOI:** 10.3390/diagnostics15101273

**Published:** 2025-05-17

**Authors:** Christos Stenos, Aikaterini Anastasiou, Georgia Nikolopoulou, Panagiotis Papanagiotou, Georgios Papagiannis, Aikaterini Koutroumpi, Danai Drakopoulou, Periklis Anastasiou, Konstantina Yiannopoulou

**Affiliations:** 1 2nd Neurological Department, Henry Dunant Hospital Center, 11526 Athens, Greece; christostenos@gmail.com (C.S.); geonikolopoulou@yahoo.gr (G.N.);; 2 Department of Neuroradiology, University Hospital Basel, 4031 Basel, Switzerland; aikaterini.anastasiou@usb.ch; 3 Department of Diagnostic and Interventional Neuroradiology, Hospital Bremen-Mitte/Bremen-Ost, 28325 Bremen, Germany; 4 1st Department of Radiology, School of Medicine, National & Kapodistrian University of Athens, Areteion Hospital, 11528 Athens, Greece; 5 Biomechanics Laboratory, Physiotherapy Department, University of the Peloponnese, 23100 Sparta, Greece; grpapagiannis@yahoo.gr; 6 Medical School, University of Ioannina, 45110 Ioannina, Greece

**Keywords:** carotid stump syndrome, acute ischemic stroke, pseudo-occlusion, internal carotid artery occlusion, carotid endarterectomy, digital subtraction angiography

## Abstract

**Background and Clinical Significance:** Carotid stump syndrome (CSS) is a rare and unexpected cause of recurrent ischemic ipsilateral events in the carotid vascular territory despite the demonstrated occlusion of the internal carotid artery (ICA). It is believed to be caused by microemboli due to turbulent blood flow in the patent stump of the occluded ICA that passes through anastomotic channels and retrograde flow into the middle cerebral artery circulation. **Case Presentation:** We describe the case of a 65-year-old male patient who suffered multiple concurrent transient ischemic attacks (TIAs) with a totally occluded ipsilateral ICA revealed by computed tomography angiography (CTA). He was diagnosed with CSS, which required the safest therapeutic approach. A further investigation with digital subtraction angiography (DSA) was performed, and a trickle of blood flow was observed in the reportedly occluded ICA. The diagnosis of a true ICA occlusion was withdrawn, and a diagnosis of pseudo-occlusion was established, affecting the final treatment strategy. Therefore, the patient underwent an ipsilateral carotid endarterectomy (CEA), and he has remained asymptomatic since then. **Conclusions:** The differentiation between a pseudo-occlusion and a true ICA occlusion is essential in promptly managing acute recurrent ipsilateral ischemic strokes in the carotid vascular territory. A further investigation with DSA in cases with a totally occluded ICA using CTA is essential for excluding pseudo-occlusions in ipsilaterally symptomatic patients.

## 1. Introduction

Carotid artery disease constitutes a major etiological factor in acute ischemic strokes (AISs) and accounts for 15–20% of all AISs [1]. In recently symptomatic carotid artery stenosis (50–99%), a recanalization of the vessel in the following 14 days will provide the maximum benefit in most patients, while CEA is the main choice of intervention [2,3,4]. However, based on the available data, carotid artery stenting (CAS) is also suggested instead of CEA for well-selected patients with recently symptomatic carotid stenosis of 50 to 99% [4]. Conversely, vascular interventions are not indicated for a total vessel occlusion [2,3].

CSS is a rare clinical entity secondary to ICA occlusions, which produces recurrent ipsilateral cerebrovascular events manifested either as transient ischemic attacks or amaurosis fugax. The underlying mechanism of this syndrome is believed to be a microembolization process from the occluded ipsilateral internal carotid artery that reaches the intracranial circulation through multiple anastomoses [5]. 

We present a case of a patient with typical CSS comprising crescendo transient ischemic attacks (TIAs) and ipsilateral total ICA occlusion in the CTA. Given the ipsilateral occurrence of multiple minor infarcts in the brain under magnetic resonance imaging (MRI), concomitant with TIA symptomatology in the same brain region, in the absence of a cardioembolic background, we proceeded to further investigate with digital subtraction angiography (DSA). The final exam revealed a trickle of blood flow in the supposedly occluded ICA. We discuss the necessity of performing DSA in cases of symptomatic occluded ICAs diagnosed by CTA and its impact on the final treatment strategy. Although an asymptomatic ICA occlusion has a relatively benign course, patients with a symptomatic pseudo-occlusion of the ICA are at high risk of recurrent thromboembolic events, and CEA might be a therapeutic choice in some cases [6]. Conversely, vascular interventions are not indicated for a total ICA occlusion. Differentiation between pseudo-occlusion and true occlusion is essential in the prompt management of acute ipsilateral ischemic stroke in the anterior circulation [6].

Although rare, CSS is a treatable cause of crescendo TIAs and stroke. However, a CSS diagnosis can only be made with well-selected imaging modalities. Our case report indicates that further investigation with DSA in cases with a totally occluded ICA in CTA is essential for excluding pseudo-occlusions due to CSS in ipsilaterally symptomatic patients.

At present, the gold standard for CSS treatment is an internal carotid artery stump surgical excision through the ipsilateral external carotid artery (ECA) endarterectomy [7].

## 2. Case Report

**Figure 1:**
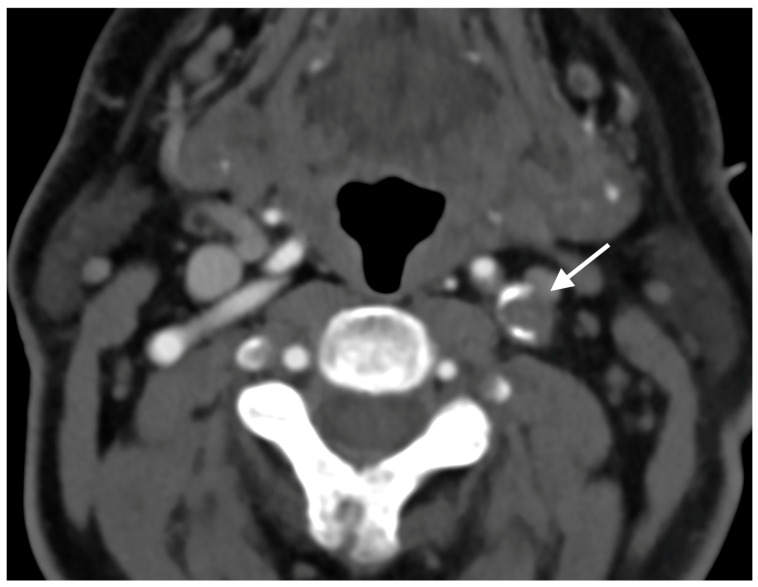
Initial CTA demonstrating complete nonattenuation of proximal cervical segment of left ICA.

**Figure 2:**
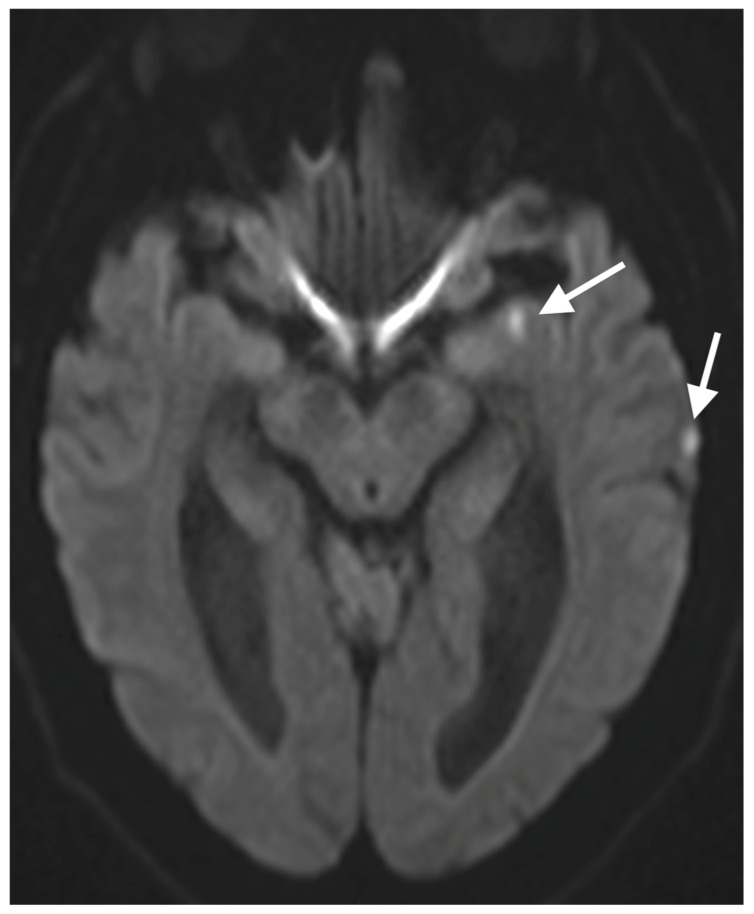
The Brain DWI MRI 24 h after the initial ischemic attack.

**Figure 3:**
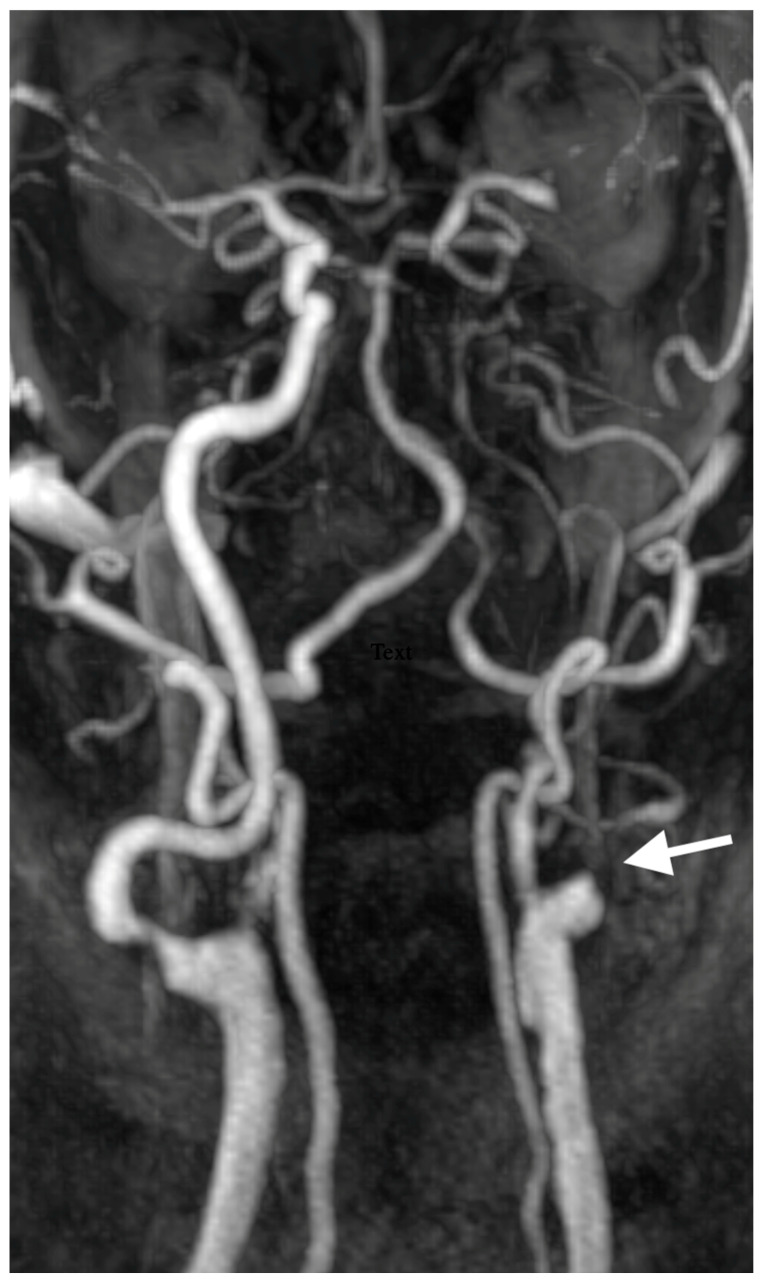
Initial MRA demonstrating complete nonattenuation of proximal cervical segment of left ICA.

**Figure 4:**
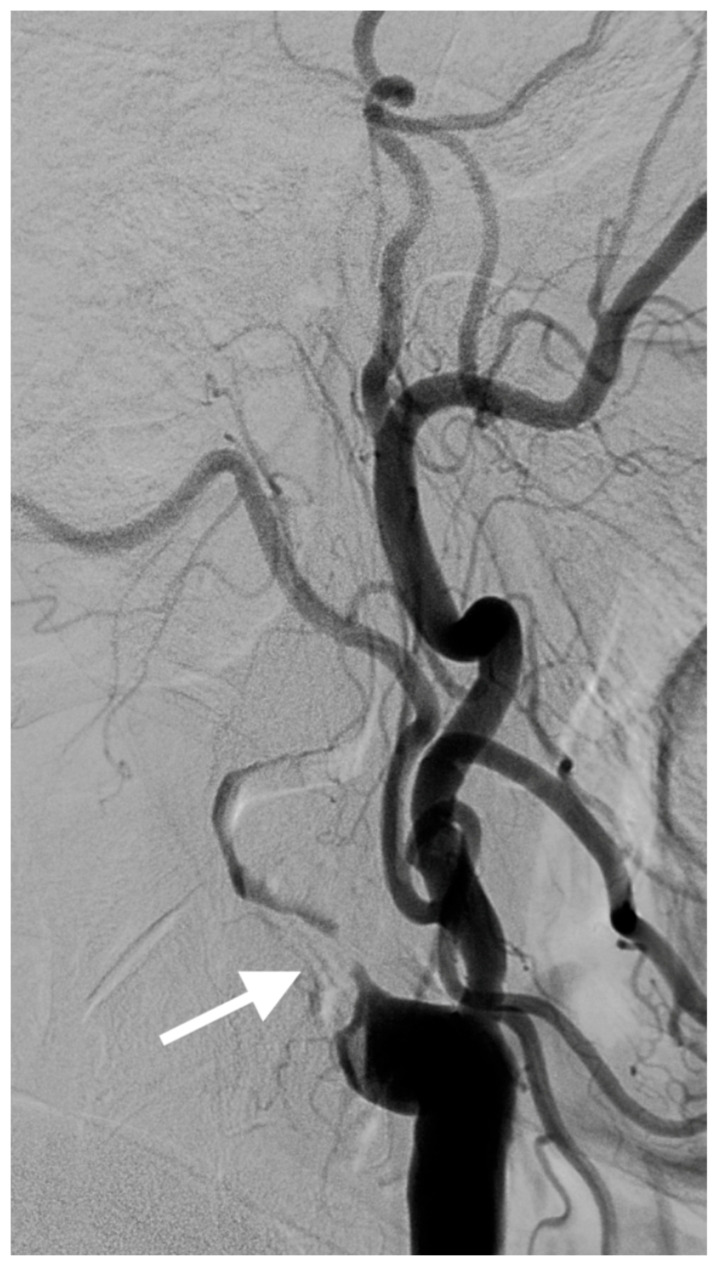
The lateral DSA with the “string sign” in the occluded left ICA before the CEA.

A 67-year-old male patient with a medical history of hypertension, coronary artery disease, and diabetes mellitus type 2 presented to the emergency department reporting an acute onset speech disturbance and weakness of the right upper limb, starting 1 h before his presentation. In the clinical examination, he had a National Institutes of Health Stroke Scale (NIHSS) score of four with mild aphasia, right central facial nerve palsy, and right upper limb paresis; however, his symptoms were completely resolved 20 min after his arrival. The patient underwent an urgent brain MRI scan, showing no acute brain infract and an MR-Angiography scan, which suggested a total left proximal ICA occlusion and a high grade (70%) occlusion of the right ICA. These findings were obtained via CTA, which was also performed urgently (Figure 1). Due to the spontaneous resolution of symptoms with an NIHSS score of 0 during and after the radiologic investigation, the possibility of intravenous thrombolysis was deferred. The patient’s symptoms were attributable to a high-risk TIA and he received clopidogrel combined with aspirin (clopidogrel 300 mg as a loading dose on day 1, followed by 75 mg daily from day 2 and aspirin 100 mg on the first day, followed by 100 mg daily) [8,9]. Afterward, the patient was transferred to the neurology department for further evaluation and continuous vital sign monitoring. During the first day of admission, he suffered further crescendo TIAs (at least six distinctive episodes) with similar symptomatology; however, no hemodynamic trigger was considered possible, raising suspicion of a thromboembolic mechanism of infarction. His NIHSS scoring during every TIA fluctuated from 1 to 4 and rapidly returned to 0 within a maximum time interval of 10 min. The cardiologic investigation with a 24 h Holter monitor and transthoracic cardiac ultrasound was negative. A new brain ΜRI study was conducted 24 h later, which demonstrated multiple ischemic lesions ipsilateral to the occluded internal carotid artery (Figure 2). These findings (negative cardiologic investigation and multiple ischemic lesions ipsilateral to the occluded ICA) raised concerns of an unstable plaque in the ipsilateral ICA on a pseudo-occlusion basis. Consequently, the necessity of performing a DSA in order to re-evaluate the carotid patency and to distinguish between a true occlusion and a possible pseudo-occlusion seemed inevitable. Contrary to the CTA and MRA results, the DSA revealed the “string sign” in the occluded ICA (Figure 1, Figure 3, and Figure 4) and confirmed the pseudo-occlusion. Although this small amount of trickle flow could not effectively perfuse the brain, emboli from the carotid stump could be transported either through the external carotid artery and the collateral circulation to the brain or through the near-occluded lumen and cause distal infarction, the so-called CSS.

Based on the ICA pseudo-occlusion imaging in the DSA and intraoperative findings, Kniemeyer et al. classified the ICA pseudo-occlusion into three types (type I: subtotal stenosis but delayed orthograde filling of the whole ICA; type II: total occlusion of the ICA at the carotid bifurcation, but delayed orthograde filling of the cervical portion and the siphon by atypical collaterals of the proximal ICA (not always angiographically detectable); and type III: no visible cervical ICA, only the petrous part and the siphon are patent due to the retrograde filling of the ICA via the Circle of Willis and ophthalmic artery) [10]. Imaging showed that this patient had a type II occlusion.

Although he was receiving a dual antiplatelet treatment, the patient suffered two further TIAs during the second and third days of admission repeating the same clinical pattern: mild aphasia; mild right-upper limb paresis; and/or mild left facial palsy with an NIHSS score of up to 3 and rapidly returning to 0 within a maximum time interval of 10 min.

Given the recurrence of multiple TIAs in the first 72 h after admission, while receiving optimal medical treatment, we believed that the patient was at high risk of a consequent major vascular event. Thus, urgent carotid revascularization became necessary [11].

**Figure 5:**
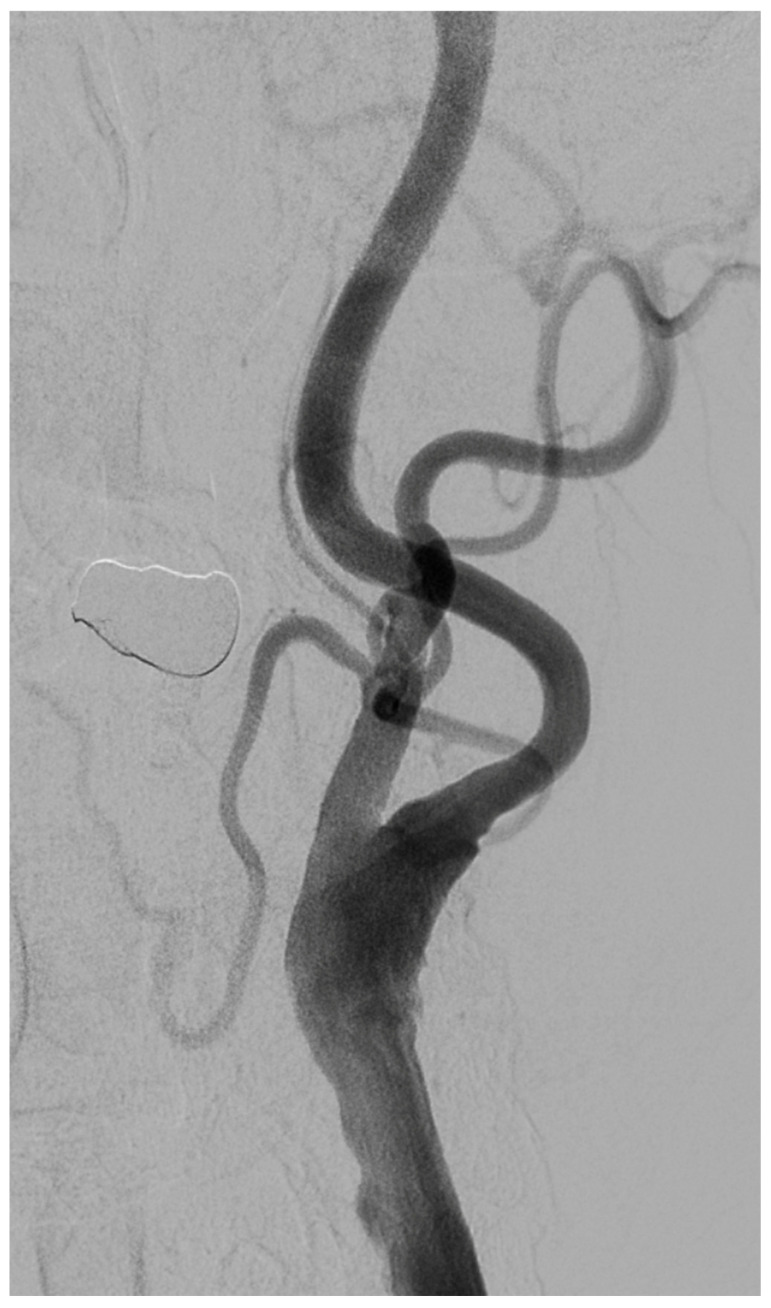
The lateral DSA after the CEA of the left ICA.

Two days later, our patient underwent a CEA of the left ICA with an optimal outcome (Figure 5). A right carotid stump endarterectomy was performed under general anesthesia. The dissection along the platysma revealed the internal carotid artery up to the bifurcation, facilitated by a self-retaining retractor. The facial vein was ligated, and loops were placed around the common carotid. Τhe ICA was entirely disconnected at the bifurcation, thereby removing the carotid stump. An incision along the stump allowed for plaque removal, followed by the verification of blood backflow within the vessel. The procedure concluded with the closure of the carotid artery in standard fashion using 5/0 and 6/0 prolene sutures, muscle layers, subcutaneous sheath, and skin, ensuring hemostasis and the integrity of the surgical site. A subsequent right CEA was initially planned, but the patient remains asymptomatic with fully controlled vascular risk parameters (diabetes, hyperlipidemia, and hypertension), and is undergoing double antiplatelet treatment with no further right ICA occlusion. A 12-month clinical follow-up revealed no stroke recurrence.

## 3. Discussion

Our patient presented with recurrent cerebrovascular events in the setting of an occluded ipsilateral ICA according to CTA findings. This raised the clinical suspicion of a pseudo-occlusion.

The clinical continuum of carotid occlusions is wide, ranging from asymptomatic to severe stroke and death. Annual incidences of mortality and stroke in patients with one-sided ICA occlusions in general are reported to be ~30%, and the risk of an ipsilateral stroke occurring in the presence of an occluded ICA is estimated at about 3–5% per year [12,13].

A reasonable question raised after revealing a total occlusion of the ipsilateral ICA is as follows: what can cause recurrent stroke in the occluded carotid territory? Recurrent strokes can be hemodynamic, thromboembolic, or of a mixed etiology, and in carotid occlusions, the perfusion in the ipsilateral hemisphere is maintained mainly by collaterals [14]. Consequently, insufficient perfusion might lead to the failure of collateralization and hemodynamic infarcts, especially in a watershed territory. Furthermore, thromboembolic infarcts can be caused by a. CSS [7]; b. contralateral carotid/aortic disease [10]; or c. the incomplete recanalization of the ipsilateral carotid enabling a trickle blood flow with emboli [10,15].

In our patient, CSS was the specific cause of the pseudo-occlusion in the CTA and of the multiple TIAs. CSS is a rare but significant clinical condition first documented in 1978 by Fields and Lemak [7]. It is characterized by persistently recurrent cerebral and retinal ischemic events occurring in patients with an ipsilateral occlusion of the internal carotid artery (ICA) [7]. When all other sources of emboli are excluded, the ipsilateral ischemic stroke that occurs due to the emboli originating from the stump of the occluded ICA is called CSS [12]. The underlying mechanism of CSS is believed to involve embolic phenomena originating from the residual stump of the ICA or the ipsilateral external carotid artery, which can lead to the migration of emboli into the middle cerebral artery territory via the external carotid artery due to the residual presence of patent external carotid-internal carotid anastomotic channels. This process can result in recurrent ischemic strokes or transient ischemic attacks (TIAs), posing a considerable risk to affected individuals [13].

CSS is an extremely unusual condition: a retrospective study reported that over 1200 carotid endarterectomies have been performed in the past decade, with only 2 treating CSS [16]. However, it has also been demonstrated that a simply obstructed ICA, even if a stump is not detected, increases the risk of an ipsilateral stroke by 3 to 5% per year [12,13,17,18]. Considering this information, we suggest that even if CSS is an extremely unusual condition, it should be quickly and accurately diagnosed and treated.

Diagnosing CSS typically involves imaging studies, including carotid a Doppler ultrasound, MRA, CTA, or DSA to assess the status of the carotid arteries and identify any residual stump [7,19]. The presence of a patent ICA stump below a completely occluded ICA is a hallmark of this syndrome. Usually, a combination of at least two of these methods is required to confirm the diagnosis, one of which should be DSA if there are persistent or recurrent symptoms [19].

Generally, a symptomatic ICA occlusion diagnosed through non-invasive means should be angiographically confirmed to differentiate between a true total occlusion and a pseudo-occlusion [10,15,19]. Patients with pseudo-occlusions are likely to benefit from carotid revascularization. Although surgical management is associated with an increased risk of complications (stroke and TIAs) compared with conventional management, at minimum, the remaining stroke risk after revascularization is likely to be reduced for symptomatic patients [6,10]. Conversely, revascularization is not an option for truly occluded ICAs. Residual lumens missed by CTA or MRA in a pseudo-occlusion deny the patient any possible beneficial carotid revascularization procedure [15].

To the best of our knowledge, only two case reports in the literature have revealed a discrepancy between a DSA result and a CTA result leading to a pseudo-occlusion diagnosis and a total occlusion diagnosis, respectively [20,21], and only one reports CSS as the final cause of the pseudo-occlusion [20]. Both studies support the necessity of the DSA and the revascularization treatment, which ceased the evolution of ischemic events in the described patients.

However, many retrospective studies and metanalyses on a series of surgical and intravascular interventions in ICAs diagnosed as occluded using CTA and MRA reveal the crucial diagnostic role of DSA in the final diagnosis and management of ICA pseudo-occlusions [6,22,23,24,25,26,27,28,29,30].

By definition, a pseudo-occlusion is a condition where non-invasive vascular imaging demonstrates a vascular occlusion, and invasive catheter angiography documents a patent vessel [26]. Although CTA is considered as a highly valuable non-invasive diagnostic tool for investigating the carotid artery pathology, several studies have demonstrated poor results in diagnosing pseudo-occlusions [27,28], with only 20% of pseudo-occlusions detected when CTAs are assessed in routine practice [29]. Diagnosing a complete ICA occlusion requires delayed imaging to exclude the slowly filling distal ICA of the near-occlusion with full collapse. In addition to DSA, this can involve a routine postcontrast head CT after CTA (which can also evaluate growing brain lesions and delayed collateral pial arteries not shown on an initial “snapshot” CTA) or multi-phased CTA [30]. Although an asymptomatic ICA occlusion has a relatively benign course, patients with a symptomatic pseudo-occlusion of the ICA are at high risk of recurrent thromboembolic events and a CEA might be a therapeutic choice in some cases [28]. Conversely, vascular interventions are not indicated for a total vessel occlusion. The differentiation between a pseudo-occlusion and a true occlusion of the ICA is essential for promptly managing an acute ipsilateral ischemic stroke of the anterior circulation. Treatment planning can be totally altered by a diagnosis of a true ICA occlusion, so it is obvious that a misdiagnosis between a total and near-total occlusion can deprive the patient of a potentially beneficial procedure.

Although limited by the scarcity of comprehensive clinical trials, treatment options for CSS have traditionally included optimal conservative medication, surgical interventions, and intravascular interventions.

Surgery may be indicated for carotid near-occlusions or pseudo-occlusions due to the potential for acute ischemic events [6,11]. Nevertheless, this patient population presents poor outcomes with lower functional independence and successful recanalization, as well as higher adverse events and mortality rates in specific metanalysis studies [6,21]. At the same time, conservative management is typically favored for asymptomatic cases [6,11]. However, if symptoms arise or if the occlusion poses an imminent threat, such as in our patient, surgery becomes a necessary option to mitigate complications, even with its associated risks [6,11]. Thus, clinical judgment prioritizes the immediate patient safety over conservative approaches in high-risk TIAs and recurrent strokes.

Open surgery involves performing an ECA endarterectomy by obliterating the ICA stump with oversewing or by placing a large metallic clip [5], while the most common approach involves ligating the ICA, which is often accompanied by an endarterectomy and the patching of the common and external carotid arteries. These procedures aim to eliminate the source of emboli and restore adequate cerebral perfusion. However, their effectiveness can vary, and the decision to proceed with surgery must be carefully weighed against the risks involved [23].

The largest study to date [23] on patients who underwent open treatments for CSS included 25 patients, all but 2 of whom remained asymptomatic at follow-up. In a pilot study comparing the best medical management and surgical treatments for patients with CSS and one symptomatic event, 10 patients were randomized to medical therapy and 15 patients to surgery [24]. One vascular event recurred in a patient who was treated by medical means, while one patient in the surgical treatment group died of myocardial infarction. To a certain extent, surgery is safe and effective, but a higher risk of perioperative complications may overshadow the clinical benefits for patients. Compensating an occluded ICA through the ipsilateral external carotid artery can be a significant risk in surgical treatment, considering the clamping and blocking of the external carotid artery during the operation and the risk of low infusion and ischemic stroke events [12].

Endovascular treatments, such as covered stents, bare stents with or without coils, and the coiling of the carotid stump, have been described in the literature. A recent systematic literature review of the endovascular treatment options for CSS [5] showed that an endovascular treatment for CSS demonstrates a low intraprocedural risk while effectively alleviating recurrent symptoms. In that systematic review, 13 patients were provided with endovascular treatments for CSS. No patients had a major intraoperative complication.

This minimally invasive approach offers a promising alternative to traditional surgical methods, reducing complications and enhancing patient outcomes. These findings underscore the efficacy of endovascular techniques in managing CSS, suggesting a shift towards these methods in clinical practice for improved patient care. Further studies may solidify these conclusions and expand treatment protocols for CSS [31].

Nevertheless, it is difficult to directly compare open versus endovascular treatments for CSS due to the low number of CSS cases and the heterogeneity in the patient population. Another confounding factor is that endovascular treatment is a continually evolving process, and the results of past treatments may not be comparable to the current treatments. For example, a recent case report of endovascular coiling as the primary treatment modality for CSS describes a treatment option that has not been previously reported in the literature [32]. This approach has the advantage of not requiring long-term antiplatelet therapy, which is unavoidable in patients receiving carotid stenting for CSS.

Overall, future studies with large cohorts are needed to make a definitive conclusion on the most optimal management. Direct comparative studies between surgical and endovascular outcomes are needed but are difficult to conduct due to the rarity of CSS.

Until then we can use the available data to manage symptomatic 99% ICA occlusions.

It is explicitly stated in randomized controlled trials that CEAs are safe and effective for reducing the risk of ischemic stroke in patients who have symptomatic ICA atherosclerosis with up to 99% stenosis [2,3] when all of the following are present: an ipsilateral TIA or nondisabling ischemic stroke (symptomatic event), a surgically suitable carotid artery lesion, no prior ipsilateral endarterectomy, and no contraindications to revascularization. In addition, the risk of perioperative stroke and death with CEAs for the surgeon or surgical center should be <6%. Concomitantly, CAS is suggested instead of CEAs for selected patients with recently symptomatic carotid stenosis of 50 to 99% who have one or more of the following: a carotid lesion unsuitable for the surgical approach; radiation-induced stenosis or carotid restenosis after an endarterectomy; clinically significant cardiac or pulmonary disease or other diseases that substantially increase the risk of anesthesia and surgery; or an unfavorable neck anatomy (e.g., contralateral vocal cord paralysis, open tracheostomy, and prior radical surgery) [25].

As endovascular treatments are an almost equally considered option for CSS treatments, we suggest that the final exclusion of a pseudo-occlusion via DSA—as well as the most accurate depiction of the anatomy of the ICA territory—is significantly important for the final therapeutic strategy approach and the clinician’s choice between surgical and endovascular interventions.

As our study is a case report of a rare clinical entity (CSS) with a limited presence in the literature, certain limitations are unavoidable. We must confess our inability to generalize our conclusions, the risk of the over-interpretation of our case, and the risk of distracting the reader with the unusual.

## 4. Conclusions

Carotid stump syndrome represents a rare but important cause of recurrent cerebral ischemic events following ICA occlusions. Understanding its pathophysiology, clinical manifestations, and treatment options is essential for healthcare providers managing patients at risk for this condition. Continued research and case reporting are necessary to redefine treatment strategies and improve outcomes for individuals affected by CSS.

Additionally, a further investigation with DSA in cases with a totally occluded ICA using CTA and/or MRA seems to be essential for excluding pseudo-occlusions in ipsilaterally symptomatic patients.

## Data Availability

No new data were created.

## Abbreviations

CSS	carotid stump syndrome
ICA	internal carotid artery
ECA	external carotid artery
CT	computed tomography
NIHSS	National Institutes of Health Stroke Scale
CTA	computed tomography angiography
DSA	digital subtraction angiography
CEA	carotid endarterectomy
AIS	acute ischemic strokes
TIA	transient ischemic attacks
MRI	magnetic resonance imaging
MRA	magnetic resonance angiography
CAS	carotid artery stenting
